# WTAP–VIRMA counteracts dsDNA binding of the m^6^A writer METTL3–METTL14 complex and maintains *N*^6^-adenosine methylation activity

**DOI:** 10.1038/s41421-023-00604-5

**Published:** 2023-10-03

**Authors:** Xuhui Yan, Feiqing Liu, Junjun Yan, Mengjun Hou, Min Sun, Delin Zhang, Zhou Gong, Xu Dong, Chun Tang, Ping Yin

**Affiliations:** 1 National Key Laboratory of Crop Genetic Improvement, Hubei Hongshan Laboratory, Huazhong Agricultural University, Wuhan, Hubei China; 2 Innovation Academy for Precision Measurement Science and Technology, Chinese Academy of Sciences, Wuhan, Hubei China; 3 College of Chemistry and Molecular Engineering, and Peking-Tsinghua Center for Life Sciences, Peking University, Beijing, China

**Keywords:** RNA modification, NMR spectroscopy

Dear Editor,

*N*^6^-methyladenosine (m^6^A) is a prevalent epigenetic modification found in eukaryotic mRNA and plays a crucial role in regulating gene expression in various physiological processes^1^. The m^6^A is installed on mammalian mRNA by a multicomponent methyltransferase complex (MTC) comprising a catalytic subunit (including METTL3 and METTL14) and a regulatory subunit (including WTAP, VIRMA, HAKAI, ZC3H13, and RBM15/15B)^2^. In the catalytic subunit, METTL3 and METTL14 form a stable heterodimer that catalyzes the addition of m^6^A to a preferred consensus RNA motif RRACH (R = A/G, H = A/C/U)^3^. WTAP and VIRMA interact with each other and mediate METTL3–METTL14 localization. Knockdown of WTAP or VIRMA causes substantial impacts on total mRNA m^6^A levels^2^. Structural and biochemical studies have revealed that a quaternary complex called M3–M14–W–V forms, which exhibits significantly higher methylation activity than the METTL3–METTL14 binary complex^4,5^. Despite these advances, how WTAP–VIRMA regulates methylation activity remains largely unknown. Here, we show that the METTL3–METTL14 complex possesses promiscuous DNA-binding activity, which disrupts its ability of RNA methylation. Moreover, WTAP–VIRMA counteracts the binding of METTL3–METTL14 to double-stranded DNA (dsDNA) and thus maintains its RNA methylation activity.

**Fig. 1:**
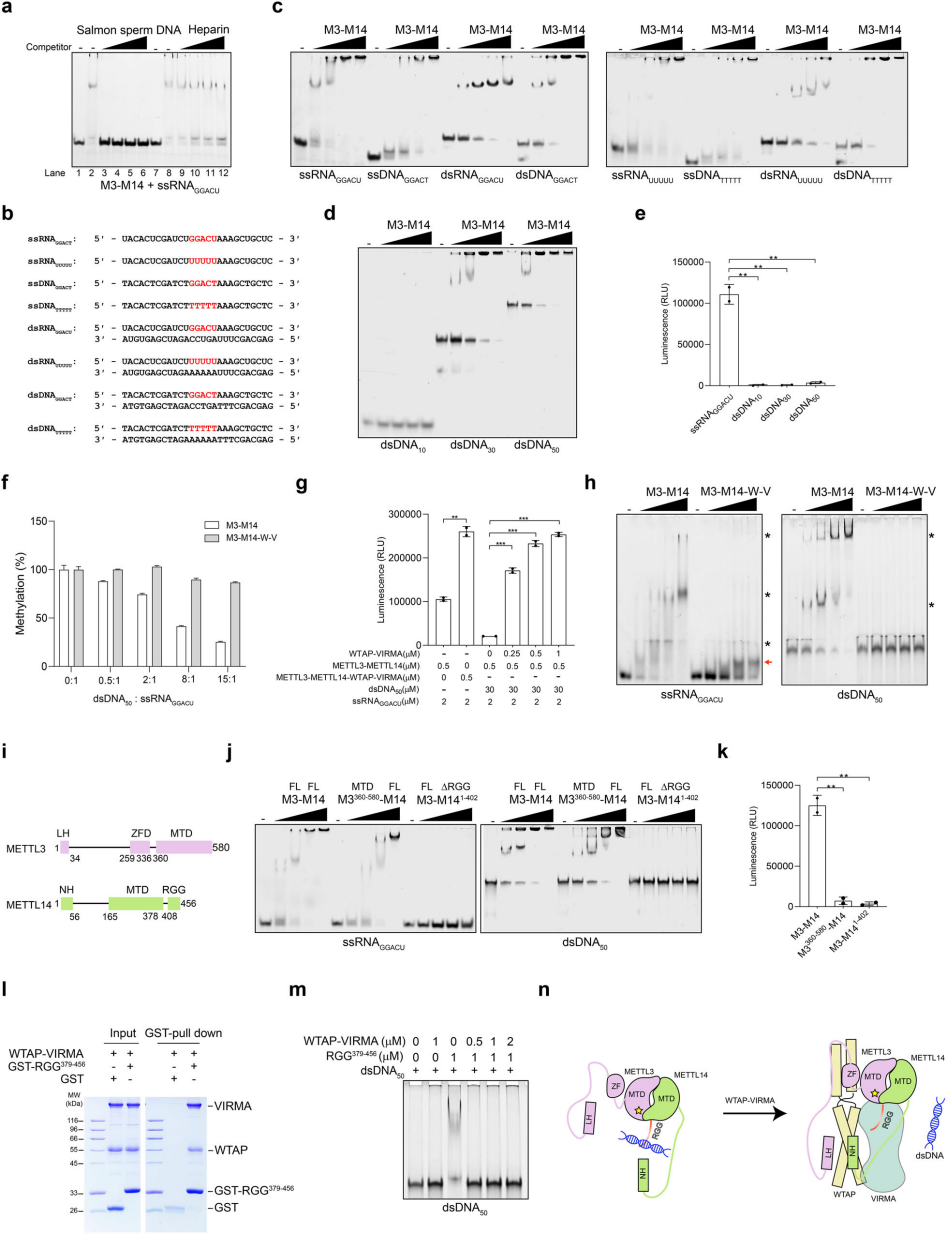
Regulatory role of WTAP–VIRMA on the m^6^A writer METTL3–METTL14 complex. **a** Binding specificity of METTL3–METTL14 (M3–M14) to ssRNA_GGACU_. Competitors were added as indicated. Lanes 3–6, salmon sperm DNA; lanes 9–12, heparin. **b** Sequences of DNA and RNA oligos used in this study. **c** Nucleic acid binding activity of the M3–M14 complex revealed by EMSA. **d** EMSA of M3–M14 binding to dsDNA probes with different lengths (10 bp, 30 bp and 50 bp). The sequences are shown in Supplementary Fig. S2b. **e** M3–M14 has no catalytic activity on dsDNA. **f** The RNA methylation activities of M3–M14 and M3–M14–W–V after a gradient concentration of dsDNA_50_ was mixed. **g** WTAP–VIRMA restricts the binding of METTL3–METTL14 to dsDNA and maintains its RNA methylation activity. **h** Nucleic acid binding ability of M3–M14 and M3–M14–W–V. The nucleic acid-bound complex is highlighted by black asterisks and red arrow. **i** Schematic diagram of the domain information of METTL3 and METTL14. ZFD, zinc finger domain; MTD, methyltransferase domain; RGG, RGG motif. **j** EMSA analysis of M3–M14 and its truncation constructs (ZFD or RGG was lacking). **k** RNA methyltransferase activity of M3–M14 and its truncation constructs. **l** GST pull-down assay between GST-RGG^379–456^ and WTAP–VIRMA. **m** WTAP–VIRMA restricts the dsDNA binding activity of RGG^379–456^. **n** The proposed model for the regulatory role of WTAP–VIRMA in the M3–M14–W–V complex. Data are shown as the means ± SD (*n* = 2).

We first investigated the binding specificity of the METTL3–METTL14 complex to a 27-nt single-stranded RNA (ssRNA) containing the GGACU modification sequence (ssRNA_GGACU_) by electrophoretic mobility shift assay (EMSA), with heparin or salmon sperm DNA as nonspecific competitors. We found that METTL3–METTL14 could efficiently bind to ssRNA_GGACU_ in the absence of nonspecific competitors or in the presence of heparin, and this binding was not observed when salmon sperm DNA was present (Fig. 1a). Considering that salmon sperm DNA is rich in DNA and heparin is a highly electronegative acidic mucopolysaccharide, we hypothesized that METTL3–METTL14 could bind to DNA. We then investigated the ability of METTL3–METTL14 to bind ssRNA/DNA and dsRNA/DNA with or without the modification sequence (Fig. 1b). The EMSA results revealed that METTL3–METTL14 could bind to four oligos containing the modification sequence (ssRNA_GGACU_, ssDNA_GGACT_, dsRNA_GGACU_, and dsDNA_GGACT_). Interestingly, substituting the modification sequence with “UUUUU” or “TTTTT” did not obviously affect their binding with METTL3–METTL14 (Fig. 1c). Moreover, METTL3–METTL14 can bind to random ssRNA/DNA probes (Supplementary Fig. S1a). Nevertheless, METTL3–METTL14 shows methylation activity only on ssRNA/DNA with the modification sequence, which is consistent with previous findings^6,7^ (Supplementary Fig. S1b). Taken together, these results suggest that METTL3–METTL14 may exhibit a promiscuous binding ability towards ssRNA/DNA and dsRNA/DNA, regardless of the presence of the consensus modification sequence.

The observation that METTL3–METTL14 binds to dsDNA attracted our attention due to the following considerations: (1) MTC can be recruited by histone modifications to deposit m^6^A co-transcriptionally^2^; (2) chromatin immunoprecipitation sequencing analysis of METTL3 and METTL14 revealed their interaction with chromatin^8^; and (3) in addition to being on RNA as methyltransferase, METTL3 and METTL14 localize to different genomic loci (such as promoters and enhancers) to regulate transcription in a m^6^A-independent manner^9^. We next examined whether METTL3–METTL14 interacts with nucleosomes, the basic repeat subunit of chromatin. METTL3–METTL14 displayed direct interaction with the reconstituted nucleosome, as well as the 147-bp Widom 601 DNA alone (Supplementary Fig. S2). Further, we found that the binding of METTL3–METTL14 to DNA diminishes as the DNA length decreases, with no observable binding to the 10-bp dsDNA_10_ (Fig. 1d). Consistently, METTL3–METTL14 exhibited no catalytic activity on dsDNA^6,7^ (Fig. 1e). These data suggest that METTL3–METTL14 can bind to dsDNA above a certain length threshold.

METTL3–METTL14 binds to DNA but does not install modifications, suggesting that DNA may influence its RNA methyltransferase activity. To test this, we measured the methyltransferase activity of METTL3–METTL14 in the presence of dsDNA_50_ at different molar ratios (dsDNA_50_/ssRNA_GGACU_: 0:1, 0.5:1, 2:1, 8:1, and 15:1). We observed a decrease in the methyltransferase activity of METTL3–METTL14 after dsDNA_50_ was added to the reaction, which was positively correlated with the amount of dsDNA_50_ added (Fig. 1f). Subsequent EMSA analysis revealed that METTL3–METTL14 displays slightly higher binding affinity to ssRNA_GGACU_ than to dsDNA_50_, and the increase of dsDNA_50_ gradually counteracts the binding of METTL3–METTL14 to ssRNA_GGACU_, especially at the dsDNA/ssRNA ratio of over 2:1 (Supplementary Fig. S3a, b). Moreover, we found that the length of DNA also impacts the methylation of METTL3–METTL14 on ssRNA. Compared to the 50-bp dsDNA_50_, the 250-bp dsDNA exhibited a much more pronounced inhibitory effect on the RNA methyltransferase activity of METTL3–METTL14 (Supplementary Fig. S3c–e).

WTAP and VIRMA have been reported as two important regulatory proteins in the MTC that impact the global mRNA m^6^A deposition in vivo, likely by recruiting METTL3–METTL14 to target RNAs^2^. We examined the methyltransferase activity of M3–M14–W–V when dsDNA_50_ was present. Intriguingly, we observed no obvious inhibitory effect of dsDNA_50_ on the methyltransferase activity of M3–M14–W–V (Fig. 1f). Moreover, M3–M14–W–V exhibited significantly higher methyltransferase activity on ssRNA_GGACU_ than METTL3–METTL14. Upon adding WTAP–VIRMA to the methylation reaction containing METTL3–METTL14, ssRNA_GGACU_ and dsDNA_50_, we observed a dose-dependent increase in the methyltransferase activity of METTL3–METTL14 (Fig. 1g). These observations led us to further investigate the binding of METTL3–METTL14 and M3–M14–W–V with three representative probes, ssRNA_GGACU_, dsDNA_50_, and ssRNA_UUUUU_. M3–M14–W–V exhibited weak binding to dsDNA_50_ compared to METTL3–METTL14 and a different pattern of binding with ssRNA (Fig. 1h, red arrow; Supplementary Fig. S4). Specifically, METTL3–METTL14 exhibited a more complex migration pattern with at least four discernible bands in EMSA (indicated by asterisks and arrow), compared to M3–M14–W–V. This complexity could arise from the promiscuous nucleic acid binding activity of METTL3–METTL14. Additionally, we found that METLL3–METTL14 can bind to different types of RNAs, including small nuclear RNA, random ssRNA, and tRNA^phe^. The presence of these RNAs inhibited the methyltransferase activity of METLL3–METTL14 on ssRNA_GGACU_. Similar to dsDNA, WTAP–VIRMA counteracts the binding of METLL3–METTL14 to these RNAs, and thus restores ssRNA methylation activity to different degrees (Supplementary Fig. S5). Taken together, these results suggest that WTAP–VIRMA plays an important regulatory role in MTC, which restricts the binding of METTL3–METTL14 to dsDNA and different types of RNA, thus maintaining the methyltransferase activity of METTL3–METTL14.

In METTL3–METTL14, two types of nucleic acid-binding domains were identified^2^, including the zinc finger domain (ZFD) in METTL3 and the RGG motif (RGG) in METTL14. We speculated that these two domains might contribute to promiscuous RNA/DNA binding of METTL3–METTL14. To test this, we generated truncated forms of METTL3–METTL14 lacking either of these two domains (Fig. 1i) and examined their ability to bind ssRNA_GGACU_ and dsDNA_50_. Compared to METTL3–METTL14, METTL3^360–580^–METTL14 lacking ZFD showed a slight decrease in binding with ssRNA_GGACU_ and dsDNA_50_ but completely lost methyltransferase activity (Fig. 1j, k). This can be explained by the fact that ZFD serves as a target recognition domain that weakly binds to the RNA substrate^2^. In contrast, METTL3–METTL14^1–402^ without the RGG could not bind nucleic acids and exhibited no methyltransferase activity (Fig. 1j, k). We then mapped the domains of METTL3–METTL14 responsible for the interaction with WTAP–VIRMA. METTL3–METTL14 could still interact with WTAP–VIRMA after N-terminal truncation of METTL3 and METTL14 (METTL3^259–580^–METTL14^111–456^) or deletion of the RGG (METTL3–METTL14^1–402^), while simultaneous deletion of these three regions abolished the interactions (Supplementary Fig. S6). The RGG motifs are clustered sequences of arginine and glycine residues commonly found in many proteins, mediating nucleic acid binding and/or protein‒protein interactions^10^. We further purified the RGG and found that it directly interacts with WTAP–VIRMA (Fig. 1l). Therefore, the RGG of METTL14 helps METTL3–METTL14 bind to nucleic acids and supports the interaction between METTL3–METTL14 and WTAP–VIRMA.

To gain more insights into the mechanisms by which WTAP–VIRMA affects the ability of METTL3–METTL14 to bind dsDNA, we first examined whether the RGG of METTL14 binds to dsDNA_50_. As expected, the RGG binds directly to dsDNA_50_. However, in the presence of WTAP–VIRMA, the RGG binding with dsDNA_50_ is disrupted, similar to METTL3–METTL14 (Fig. 1m). Next, we explored the binding interfaces of the RGG with its potential interactors using nuclear magnetic resonance (NMR) assays. Initially, ^15^N-labeled RGG was examined using a ^1^H-^15^N HSQC spectrum, and the resultant spectrum showed that all amide backbone signals appeared at ~8.0 ppm, meaning a mostly disordered conformation. Subsequently, we investigated the interaction of the RGG with WTAP–VIRMA^381–1486^ (Supplementary Fig. S7), ssRNA_GGACU_, ssRNA_UUUUU_ and dsDNA_50_ using NMR titration experiments. Upon addition of the unlabeled WTAP–VIRMA^381–1486^, ssRNA_GGACU_, ssRNA_UUUUU_, or dsDNA_50_ to the ^15^N-labeled RGG, a similar set of peaks were found to be perturbed, meaning that WTAP–VIRMA^381–1486^, dsDNA_50_, ssRNA_GGACU_, and ssRNA_UUUUU_ interact with the RGG of METTL3–METTL14 via a similar interface, which may lead to their competitive binding to METTL3–METTL14. Moreover, the chemical shift perturbations were the largest for WTAP–VIRMA^381–1486^ and the smallest for ssRNA (Supplementary Fig. S8). Thus, the interaction of WTAP–VIRMA with the RGG of METTL14 likely outcompetes the binding of dsDNA to METTL3–METTL14.

In summary, we identified a novel mechanism by which WTAP–VIRMA regulates the methyltransferase activity of METTL3–METTL14 by restricting the promiscuous dsDNA-binding ability of the METTL14 RGG motif, thus maintaining RNA methylation. Specifically, METTL3–METTL14 can bind to both RNA and dsDNA when the RGG motif is present (Fig. 1c). However, the presence of dsDNA greatly reduced the RNA methyltransferase activity of METTL3–METTL14 (Fig. 1f). As the regulatory subunit, WTAP–VIRMA directly interacts with the RGG motif, prevents the binding of dsDNA, and consequently maintains RNA methylation activity of METTL3–METTL14 (Fig. 1n). Recent studies have demonstrated that METTL3 and METTL14 bind to chromatin and perform m^6^A-dependent or -independent regulatory functions at active gene transcription start sites^8,9^. The regulated recruitment of METTL3–METTL14 by WTAP–VIRMA may influence the occurrence of m^6^A-dependent and -independent functional events. Our study provides a regulatory framework for further studies on the versatile functions of the m^6^A writer complex.

### Supplementary information

Supplementary information
