# Supplementary material for: WTAP–VIRMA counteracts dsDNA binding of the m6A writer METTL3–METTL14 complex and maintains N6-adenosine methylation activity
Source: Cell Discov. 2023 Oct 3;9:100. doi: 10.1038/s41421-023-00604-5 (PMC10547700; doi:10.1038/s41421-023-00604-5)
Supplement: Supplementary file 1 — Supplementary information [file 41421_2023_604_MOESM1_ESM.pdf]

WTAP-VIRMA counteracts dsDNA binding of the m^{6}A writer METTL3-METTL14 complex and maintains N^6-adenosine methylation activity

Xuhui Yan[1,4], Feiqing Liu[1,4], Junjun Yan[1], Mengjun Hou[1], Min Sun[2], Delin Zhang[1], Zhou Gong[2], Xu Dong[2], Chun Tang[3,*], and Ping Yin[1,*]

[1]National Key Laboratory of Crop Genetic Improvement, Hubei Hongshan Laboratory, Huazhong Agricultural University, Wuhan, Hubei, China.

[2]CAS Key Laboratory of Magnetic Resonance in Biological Systems, State Key Laboratory of Magnetic Resonance and Atomic Molecular Physics, National Center for Magnetic Resonance at Wuhan, Innovation Academy for Precision Measurement Science and Technology, Chinese Academy of Sciences, Wuhan, Hubei, China.

[3]Beijing National Laboratory for Molecular Sciences, College of Chemistry and Molecular Engineering, and Peking-Tsinghua Center for Life Sciences, Peking University, Beijing, China.

*Correspondence:
Chun Tang (Tang_Chun@pku.edu.cn) or Ping Yin (yinping@mail.hzau.edu.cn)

[4]These authors contributed equally: Xuhui Yan, Feiqing Liu

Supplementary information

Material and Methods

Molecular cloning and protein expression

Full-length *METTL3*, *METTL14*, *WTAP*, *VIRMA* and histones (*H2A*, *H2B*, *H3.2* and *H4*) were amplified from the *Homo sapiens* cDNA library. The truncated genes were subcloned using standard polymerase chain reaction (PCR) methods. The plasmid of 147-bp Widom 601 DNA was a gift from Zhanxin Wang (Beijing Normal University).

For the electrophoretic mobility shift assays (EMSAs) and the methyltransferase assays, the cDNAs of *METTL3* and *METTL14* were subcloned into pFastBac dual. *WTAP*, *VIRMA*, *METTL3*, *METTL3*$^{360-580}$, *METTL14* and *METTL14*$^{1-402}$ were subcloned into a modified pFastBac1 vector, respectively. *WTAP*, *METTL14* and *METTL14*$^{1-402}$ were fused with an N-terminal His-tag. Baculoviruses were generated in *Spodoptera frugiperda* Sf9 cells (Gibco, 12659017) with the Bac-to-Bac system (Invitrogen). The proteins were co-expressed in Sf9 cells at 27°C for 60 h before harvesting.

For histone octamer assembly, *H2A*, *H2B*, *H3.2* and *H4* were subcloned into the pQlink vector[1]. *H2A* and *H3.2* were fused with an N-terminal His-tag. The plasmid was transformed into *E. coli* BL21 (DE3) for protein expression. One-litre lysogeny broth medium supplemented with 100 μg ml^{-1} ampicillin was inoculated with a transformed bacterial preculture and shaken at 37°C until the optical density at 600 nm reached 1.0. After being induced with 0.2 mM isopropyl-β-D-thiogalactoside (IPTG), the cells were grown at 37°C for 4.5 h before harvesting.

For GST pull-down assays, plasmid 1 (METTL3 or METTL3$^{259-580}$) was subcloned into pPGH (Novagen) and fused with a GST-tag at the N-terminus. Plasmid 2 (METTL14, METTL14$^{1-402}$, METTL14$^{111-456}$ or METTL14$^{111-402}$) was subcloned into pBB75 (Novagen) without any tag. To construct the METTL3–METTL14 complex and its truncated forms, plasmids 1 and 2 were then co-transformed into *E. coli* BL21 (DE3). One-litre lysogeny broth medium supplemented

with 100 μg ml^{-1} ampicillin and 50 μg ml^{-1} kanamycin was inoculated with a transformed bacterial preculture and shaken at 37°C until the optical density at 600 nm reached 1.0. After being induced with 0.2 mM IPTG and grown at 16°C for 14 h before harvesting, $RGG^{379-456}$ (also called $METTL14^{379-456}$) was subcloned into pPGH and expressed in *E. coli* BL21 (DE3) as described above.

For NMR titrations, DNA from human WTAP and $VIRMA^{381-1486}$ were subcloned into pFastBac dual. WTAP was expressed with an N-terminal His-tag and expressed in sf9 as described above. $RGG^{379-456}$ was subcloned into pET15d (Novagen) with an N-terminal His-tag. After transformation into *E. coli* BL21 (DE3), the cells were grown in M9-minimum medium with $^{15}NH_4Cl$ (Isotec, Miamisburg, OH) as the sole nitrogen source for preparing isotope-enriched protein. Protein expression was induced with 0.5 mM IPTG at an OD_{600nm} of 1.0. After induction, the culture temperature was lowered from 37°C to 20°C, and the cells were grown for another 16 h.

Protein purification

The sf9 cells were harvested by centrifugation at 1500 × g for 15 minutes and homogenized in ice-cold lysis buffer (25 mM Tris-HCl, pH 8.0 and 150 mM NaCl) with 1 mM phenylmethanesulfonyl-fluoride (PMSF). The supernatants were loaded onto Ni-NTA resin and washed three times with high salt buffer (25 mM Tris-HCl, pH 8.0 and 1 M NaCl) and one time with lysis buffer. Elution was performed in elution buffer (25 mM Tris-HCl, 250 mM imidazole, pH 8.0 and 2 mM dithiothreitol (DTT)) and applied to a Source Q10/100 column (GE Healthcare), followed by gradient NaCl elution (up to 1 M) in 25 mM Tris–HCl (pH 8.0). Target proteins were further purified on a Superose 6 increase 10/300 GL column (GE Healthcare) equilibrated with SEC buffer (25 mM Tris-HCl, pH 8.0 and 150 mM NaCl). To acquire the METTL3-METTL14-WTAP-VIRMA complex, METTL3-METTL14 protein and WTAP-VIRMA protein were mixed at a molar ratio of 1: 0.9 at 4°C for 30 min. Then, the mixture was applied to a Superose 6 increase 10/300 column (GE Healthcare) equilibrated with SEC buffer.

For histone octamer assembly, the bacterial pellet was resuspended in histone lysis buffer (20

mM Tris-HCl, pH 8.0, 2.0 M NaCl, 25 mM imidazole and 10% glycerol) with 1 mM PMSF. After centrifugation at 14,000 rpm at 4°C, the supernatant was loaded onto Ni-NTA resin and washed three times with histone wash buffer (20 mM Tris-HCl, pH 8.0, 2.0 M NaCl, 50 mM imidazole and 10% glycerol). Bound proteins were eluted with histone elution buffer (20 mM Tris-HCl, pH 8.0, 2.0 M NaCl, 250 mM imidazole and 10% glycerol) and applied to a Hitrap Heparin HP column (GE Healthcare), followed by gradient NaCl elution (up to 2 M) in 25 mM Tris–HCl (pH 8.0). The protein sample was subsequently loaded onto a Superdex 200 increase 10/300 column (GE Healthcare) preequilibrated with histone SEC buffer (10 mM Tris-HCl, pH 8.0, 2 M NaCl and 5 mM DTT).

For nucleosome assembly, the 147-bp Widom 601 DNA sequence[2] for nucleosome reconstitution was purified as described[3]. Nucleosomes were prepared by mixing histone octamers and purified 147-bp Widom 601-containing DNAs in histone SEC buffer followed by salt gradient dilution for approximately 30 h. Pure nucleosomes were passed through a Superose 6 increase 10/300 column equilibrated with 50 mM HEPES, pH 7.5 and 50 mM NaCl for separation.

For GST pull-down assays, the bacterial pellet was collected and homogenized in high salt buffer containing 1 mM PMSF. After centrifugation at 14,000 rpm at 4°C, the supernatant was loaded onto glutathione Sepharose, washed three times with lysis buffer, and eluted with GST elution buffer (25 mM Tris-HCl, pH 9.0, 300 mM NaCl and 10 mM reduced glutathione). The elution was diluted three times and applied to the Hitrap Heparin HP column (for the $RGG^{379-456}$ protein) or Source Q10/100 column. Target proteins were subjected to the Hitrap desalting column, which was equilibrated with SEC buffer with 2 mM DTT.

For NMR titrations, the pellet was collected in ice-cold lysis buffer. The cells were disrupted in a cell homogenizer, and the insoluble fraction was removed by centrifugation at 14,000 rpm and 4°C for 1 h. The supernatant was loaded onto a gravity column of Ni-NTA resin. The resin was washed three times with high salt buffer and one time with lysis buffer. The protein was then eluted with elution buffer. The protein was further purified with the Hitrap Heparin HP

column. The His-tag was removed with drICE protease (protein and protease mixed at 100:1 molar ratio) at 25°C for 2 h. With the tag removed, the protein was purified with a Superdex 200 Increase 10/300 column with 25 mM Tris-HCl, pH 7.0, and 150 mM NaCl.

Electrophoretic mobility shift assay (EMSA)

The 6-Carboxyfluorescein (FAM)-labelled oligonucleotides and unlabelled oligonucleotides were synthesized (General Biol) and dissolved in annealing buffer (10 mM Tris-HCl, pH 8.0, 50 mM NaCl and 1 mM $MgCl_2$). The double-stranded DNA (dsDNA) and double-stranded RNA (dsRNA) were annealed by incubation in boiled water and gradually cooled to room temperature. M3-$dsDNA_{50}$ is the 50-bp sequence of METTL3 cDNA, and was annealed by incubation in boiled water and gradually cooled to room temperature. A 250 bp DNA gene (M3-$dsDNA_{250}$) amplification product generated from a plasmid encoding human METTL3, using sequence specific forward and reverse primers. The human phenylalanine tRNA ($tRNA^{phe}$) was synthesized by in vitro T7 RNA polymerase run-off transcription as described previously[4], and was refolded by rapid heating at 95°C for 3 min and slow cooling to 25°C.

To test the appropriate type and concentration of the competitor, FAM-RNA_{GGACU} (10 nM) was incubated with 0.5 μM METTL3-METTL14 on ice for 30 min in EMSA buffer (50 mM HEPES, pH 7.5, 50 mM NaCl, 50 μM $MgCl_2$ and 10% glycerol) with no competitor, salmon sperm DNA (12.5, 25, 50, and 100 μg ml^{-1}) or heparin (100, 200, 400, and 800 ng ml^{-1}). To investigate the interaction between nucleic acids and proteins, FAM-labelled oligonucleotides (10 nM), unlabelled dsDNA oligonucleotides (0.5 μM $dsDNA_{50}$ or $dsDNA_{147}$) or unlabelled tRNA were incubated with 0.25, 0.5, 1, and 2 μM proteins on ice for 30 min in EMSA buffer with 200 ng ml^{-1} heparin. To examine the impact of $dsDNA_{50}$ on the binding of METTL3-METTL14 to $ssRNA_{GGACU}$, FAM-labelled $ssRNA_{GGACU}$ (0.5 μM) and 1.5 μM METTL3-METTL14 were incubated with 0, 0.25, 1, 4, 7.5, 15, and 30 μM unlabelled $dsDNA_{50}$ oligonucleotides on ice for 30 min in EMSA buffer with 200 ng ml^{-1} heparin. The reactions were resolved on 10% or 6% native acrylamide gels (37.5:1 acrylamide: bis-acrylamide). Images of the gels with FAM-labelled oligonucleotides were obtained using Amersham Typhoon (GE Healthcare). The Gels with unlabelled dsDNA or $tRNA^{phe}$ were extracted and stained by GelRed and visualized by

Image Lab (Bio-Rad).

To estimate the equilibrium dissociation-binding constants (K_d) value, an aliquot of 10 nM FAM-labeled ssRNA$_{GGACU}$ or dsDNA$_{50}$ was mixed with sequentially diluted concentrations of METTL3-METTL14 by 3/4 folds for a total of 11 gradients (2.5 μM as initial concentration). The binding proportions were calculated by measuring the reduction of free probe. Free RNA was quantified using ImageJ. Binding curves were fitted individually using "One site–Specific binding with Hill slope" in GraphPad Prism 8.0 software. Curves were normalized to the percentage of bound oligonucleotides. The data were expressed as and the means ± SD of the K_d from two independent experiments.

For the nucleosome gel shift assay, recombinant nucleosome (2, 4, 6, 8, and 12 μM) was mixed with METTL3-METTL14 protein (1.2 μM) in EMSA buffer and incubated on ice for 30 min. The reactions were resolved on 6% native acrylamide gels (37.5:1 acrylamide: bis-acrylamide) in a 0.2 × Tris-Borate-EDTA acid buffer. Gels were extracted and stained by GelRed and visualized by Image Lab.

Methyltransferase assays

The RNA$_{GGACU}$ (5′-UACACUCGAUCUGGACUAAAGCUGCUC-3′)[5] and the RNA$_{UUUUU}$ (5′-UACACUCGAUCUUUUUUAAAGCUGCUC-3′, as negative control) were synthesized using ABI-3400 Synthesizer with phosphoramidites as previously described[6]. The sequences of the oligonucleotides used to form the dsDNA were synthesized (Tsingke), annealed through incubation in boiled water and gradually cooled to room temperature in annealing buffer.

The reactions for each enzyme were conducted under the same steady state conditions with [S]=2 μM, [SAM]=5 μM, and [E]=0.5 μM. To initiate the reactions (20 min at 25°C), substrate was added into a reaction mixture of enzyme and SAM in H buffer (50 mM HEPES, pH 7.5, 50 mM NaCl, and 50 μM $MgCl_2$). To examine the impact of different dsDNA lengths on METTL3-METTL14's methylation activity, the reaction mixture added M3-dsDNA$_{50}$ (0.4, 2.4, 6 and 12 μg ml^{-1}) or M3-dsDNA$_{250}$ (0.4, 0.8, 1.2 and 2.4 ng ml^{-1}), respectively. To investigate

the methyltransferase activity of METTL3-METTL14 and METTL3-METTL14-WTAP-VIRMA on $ssRNA_{GGACU}$ in the presence of $dsDNA_{50}$, $dsDNA_{50}$ was added to the reaction mixture to final concentrations of 0, 1, 4, 16, and 30 μM, respectively. To test the methyltransferase activity of METTL3-METTL14 and METTL3-METTL14-WTAP-VIRMA on $ssRNA_{GGACU}$ in the presence of different types of RNA (small nuclear RNA (snRNA)[7], random RNA ($ssRNA_{random}$) and $tRNA^{phe}$), these RNA probes were added to the reaction mixture to final concentrations of 8 μM, respectively. The reactions were terminated by the addition of trifluoroacetic acid (TFA) to a final concentration of 0.1% (v/v), and an 8-μl mixture was transferred to a half-area 384-well plate. The activity was measured using an MTase-Glo™ Methyltransferase Assay kit in which the reaction byproduct SAH is converted into ATP in a two-step reaction, and ATP can be detected through a luciferase reaction[8]. The luminescence signal was measured by a TECAN infinite M200 (TECAN).

GST pull-down assays

GST pull-down assays were performed to detect protein–protein interactions using GST-tagged proteins and His-WTAP-VIRMA (or His-WTAP-$VIRMA^{381-1486}$). First, GST-tagged proteins and His-WTAP-VIRMA (or His-WTAP-$VIRMA^{381-1486}$) were mixed and incubated with 50 μl GST beads in a total volume of 500 μl in SEC buffer at 4°C for 2 h with gentle rotation. Then, the supernatant of the mixture was removed, and the beads were washed three times using lysis buffer. Elution was performed using GST elution buffer, followed by SDS–PAGE analysis.

NMR experiments

All NMR experiments were recorded at 298 K using Bruker 800 MHz and Bruker 850 MHz spectrometers equipped with cryogenic probes. The NMR samples were prepared in NMR buffer (25 mM Tris-HCl, pH 7.0, 150 mM NaCl and 10% D_2O). To conduct the NMR titration experiments, WTAP-VIRMA, $ssRNA_{GGACU}$, $dsDNA_{50}$ or $ssRNA_{UUUUU}$ (25 μM) was added into ^{15}N-labelled RGG (50 μM). All NMR data were processed using NMRPipe (Version 2020) and analysed using CcpNmr analysis (Version 2.4.2).

References

1. Scheich C. et al. Vectors for co-expression of an unrestricted number of proteins. *Nucleic Acids Res.* **35**(6), e43 (2007).

2. Lowary P.T. & Widom J. New DNA sequence rules for high affinity binding to histone octamer and sequence-directed nucleosome positioning. *J. Mol. Biol*. **276**, 19-42 (1998).

3. Dyer, P. N. et al. Reconstitution of nucleosome core particles from recombinant histones and DNA. *Methods Enzymol*. **375**, 23-44 (2004).

4. Jin, X. et al. Structural insight into how WDR4 promotes the tRNA N7-methylguanosine methyltransferase activity of METTL1. *Cell Discov.* **9**, 65 (2023).

5. Liu J. et al. A METTL3-METTL14 complex mediates mammalian nuclear RNA N-6-adenosine methylation. *Nat. Chem. Biol*. **10**(2), 93-95 (2014).

6. Huang J., Dong X., Gong Z. et al. Solution structure of the RNA recognition domain of METTL3-METTL14 N(6)-methyladenosine methyltransferase. *Protein Cell*. **10**(4), 272-284 (2018).

7. Doxtader K.A. et al. Structural Basis for Regulation of METTL16, an S-Adenosylmethionine Homeostasis Factor. *Mol Cell*. **71**(6), 1001-1011.e4 (2018).

8. Hsiao K., Zegzouti H., & Goueli S.A. Methyltransferase-Glo: a universal, bioluminescent and homogenous assay for monitoring all classes of methyltransferases. *Epigenomics*. **8**, 321-339 (2016).

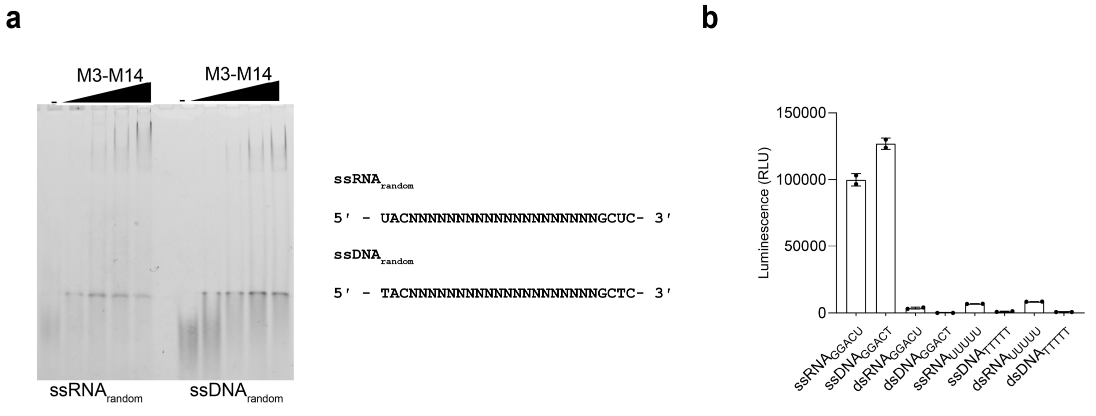

Fig. S1 Promiscuous binding activity of the METTL3-METTL14 complex. a Nucleic acid binding activity of the M3-M14 complex revealed by EMSA. Sequences of 27-bp FAM-labeled random probes are shown on the left. Due to the technical limitations, random sequences only containing 20 nt lengths are synthesized. The final concentration of oligos is 10 nM, and the final concentrations of M3-M14 are 0.25, 0.5, 1, and 2 μM. **b** METTL3-METTL14 activity on ssRNA/DNA and dsRNA/DNA. [oligo]= 2 μM, [SAM]= 5 μM, [E]= 0.5 μM. Data are shown as the means ± SD (n= 2).

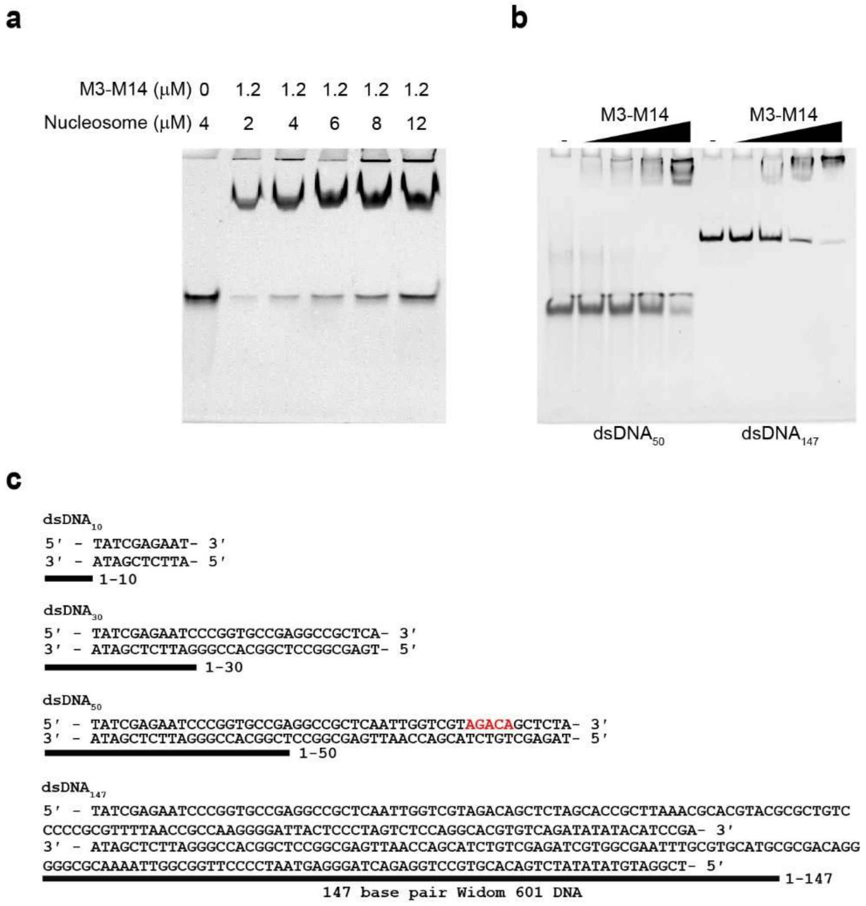

Fig. S2 Binding of METTL3-METTL14 to the nucleosome and dsDNA. a, b EMSA analysis for the interaction between METTL3-METTL14 (M3-M14) and the nucleosome (**a**) and 147-bp Widom 601 DNA (**b**). The final concentration of oligos (dsDNA) is 0.5 μM, and the final concentrations of proteins are 0.25, 0.5, 1 and 2 μM, respectively. **c** Sequences of various lengths of 147-bp Widom 601 DNA used in this study. The 147-bp Widom 601 DNA exhibits high affinity for histone octamers and is useful for assembling nucleosomes in vitro. The Gels were stained with GelRed.

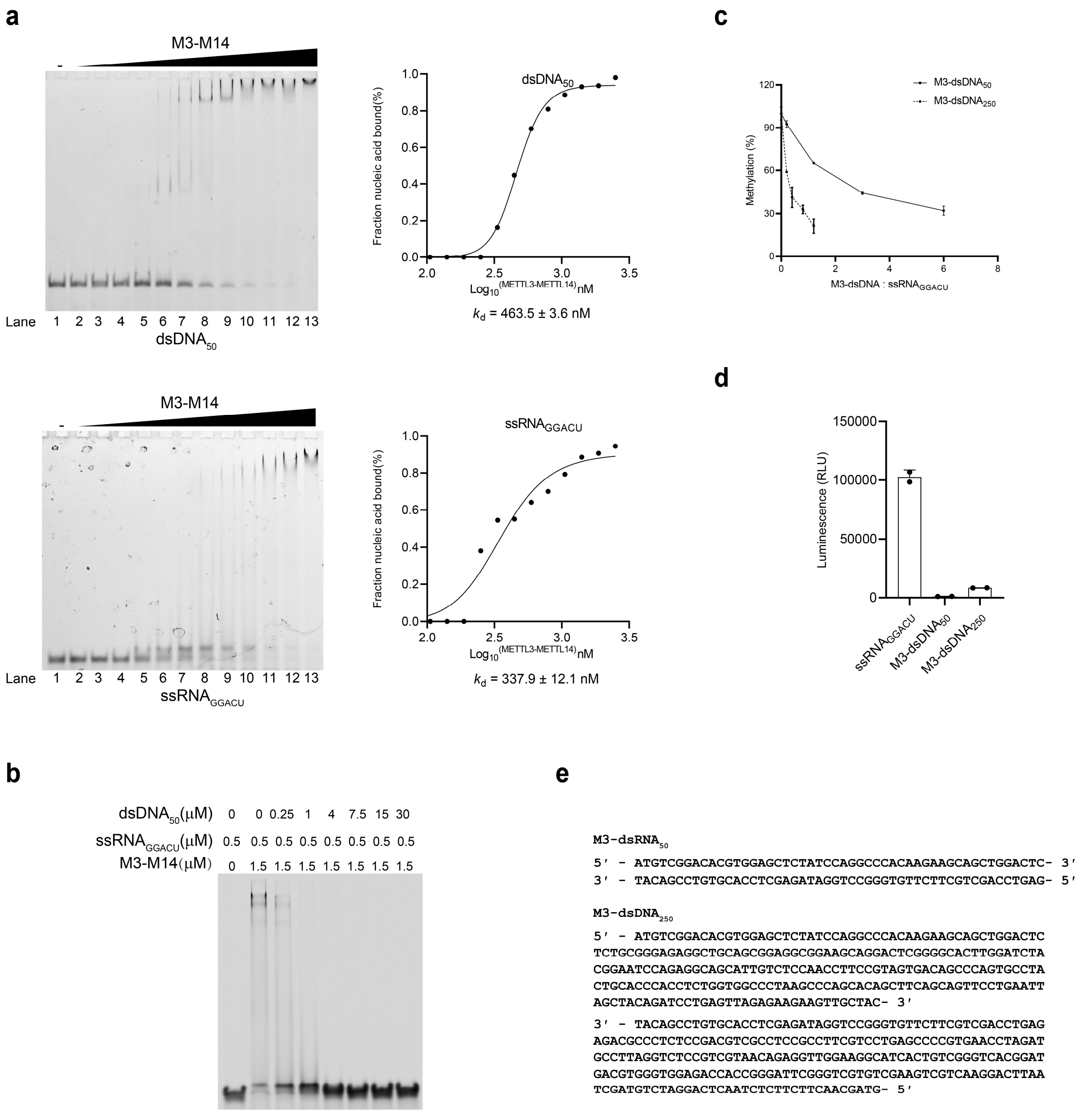

Fig. S3 Effects of dsDNA on ssRNA binding and catalytic activity of METTL3-METTL14. **a** Binding affinity of METTL3-METTL14 to $dsDNA_{50}$ (top) and $ssRNA_{GGACU}$ (bottom) is estimated as a K_d value of 463.5 ± 3.6 nM and 337.9 ± 12.1 nM, respectively. The final concentration of oligos was 10 nM. The protein concentration in lane 13 is the highest (2.5 μM), and it is subjected to a sequential dilution from lane 12 to lane 2 by 3/4-fold gradient. **b** Impact of $dsDNA_{50}$ (non-labeled) on the binding of M3-M14 to $ssRNA_{GGACU}$ (FAM-labeled). **c** RNA methylation activity of M3-M14 after addition of a gradient concentration of dsDNA. M3-$dsDNA_{50}$ and M3-$dsDNA_{250}$ are the 50-bp and 250-bp sequences of METTL3 cDNA, respectively. The molar ratios of M3-$dsDNA_{50}$ to $ssRNA_{GGACU}$ are 0/1, 0.2/1, 1.2/1, 3/1, and 6/1, and the molar ratios of M3-$dsDNA_{250}$ to $ssRNA_{GGACU}$ are 0/1, 0.2/1, 0.4/1, 0.8/1, and 1.2/1.

d M3-M14 exhibits no catalytic activity on dsDNA. [oligo]= 2 μM, [SAM]=5 μM, [E]=0.5 μM. Data are shown as the means ± SD (n= 2). **e** Sequence of the oligos have been used in **c** and **d.**

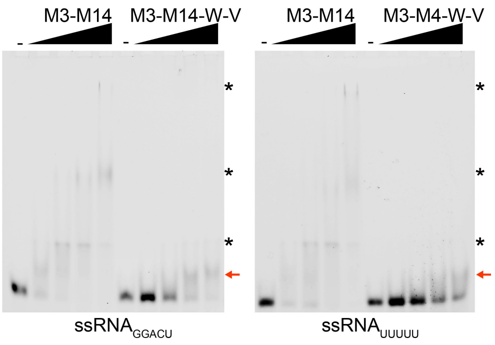

Fig. S4 Binding ability of METTL3-METTL14 and METTL3-METTL14-WTAP-VIRMA to substrate and non-substrate RNA. The final concentration of oligos was 10 nM, and the final concentrations of proteins are 0.25, 0.5, 1 and 2 μM, respectively. The nucleic acid-bound complex is highlighted by black asterisks and red arrow.

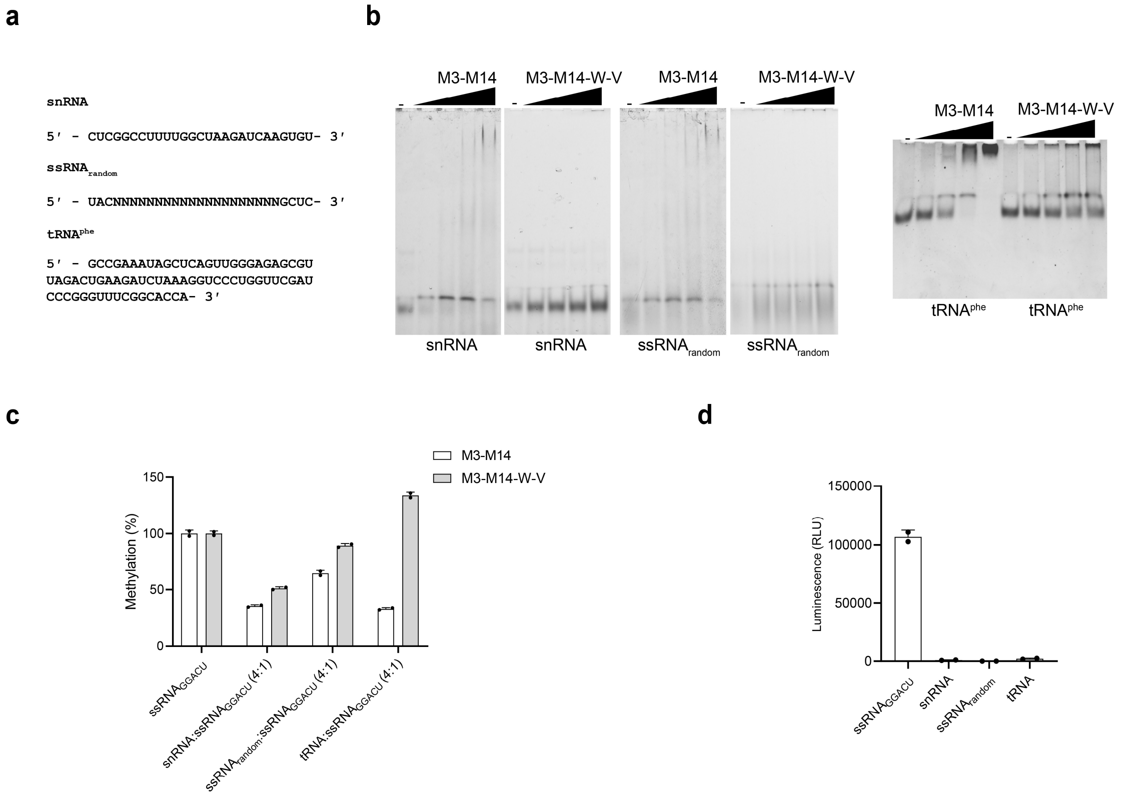

Fig. S5 WTAP-VIRMA counteracts RNA binding of m^{6}A writer METTL3-METTL14 complex and maintains N^6-adenosine methylation activity. a Sequences of the snRNA[8], ssRNA$_{random}$ and tRNA^{phe}. The snRNA and ssRNA$_{random}$ contain a covalently attached FAM. **b** Nucleic acid binding ability of M3-M14 and M3-M14-W-V. The Gels of snRNA and ssRNA$_{random}$ were scanned using the Amersham Typhoon. The Gel of tRNA^{phe} was stained with GelRed and visualized by Image Lab (Bio-Rad). The final concentration of the snRNA, ssRNA$_{random}$ and tRNA^{phe} is 10 nM, 10 nM, and 0.5 μM, respectively. The final concentrations of proteins are 0.25, 0.5, 1, and 2 μM. **c** The RNA methylation activity of M3-M14 and M3-M14-W-V after addition of RNA probe (snRNA, ssRNA$_{random}$ or tRNA^{phe}). The molar ratio of the RNA oligo (snRNA, ssRNA$_{random}$ or tRNA^{phe}) to ssRNA$_{GGACU}$ is 4/1. [ssRNA$_{GGACU}$]= 2 μM, [SAM]=5 μM, [E]=0.5 μM. **d** M3-M14 cannot catalyze the designed RNA probes. [oligo]= 2 μM, [SAM]=5 μM, [E]=0.5 μM. Data are shown as the means ±SD (n= 2).

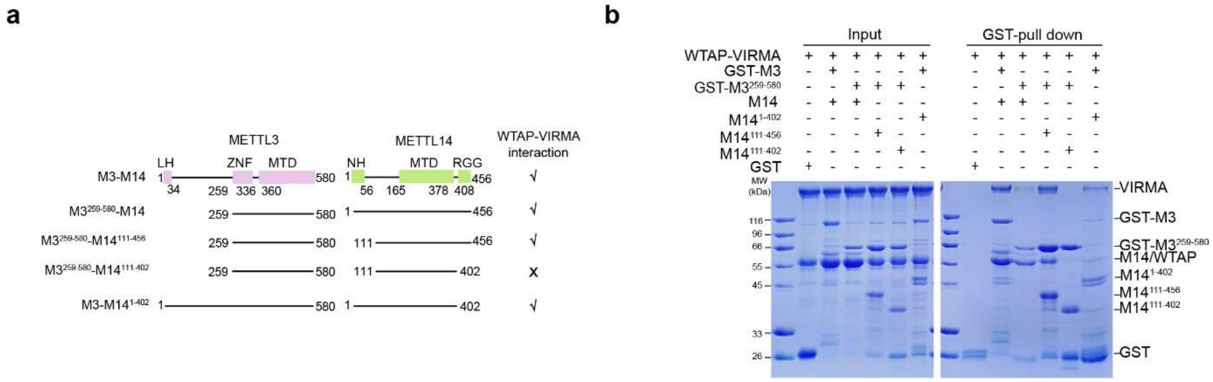

Fig. S6 The RGG motif of METTL14 contributes to the interaction between METTL3-METTL14 and WTAP-VIRMA. a Schematic view of different METTL3-METTL14 truncation constructs. LH, leader helix; ZFD, zinc finger domain; MTD, methyltransferase domain; NH, N-terminus helix; RGG, the RGG motif. **b** The GST pull-down assay between GST-METTL3-METTL14 (full length or truncations) and WTAP-VIRMA. **c** Schematic diagram of the domain information of WTAP and VIRMA. **d** The GST pull-down assay between GST-RGG$^{379\text{-}456}$ and WTAP-VIRMA$^{381\text{-}1486}$.

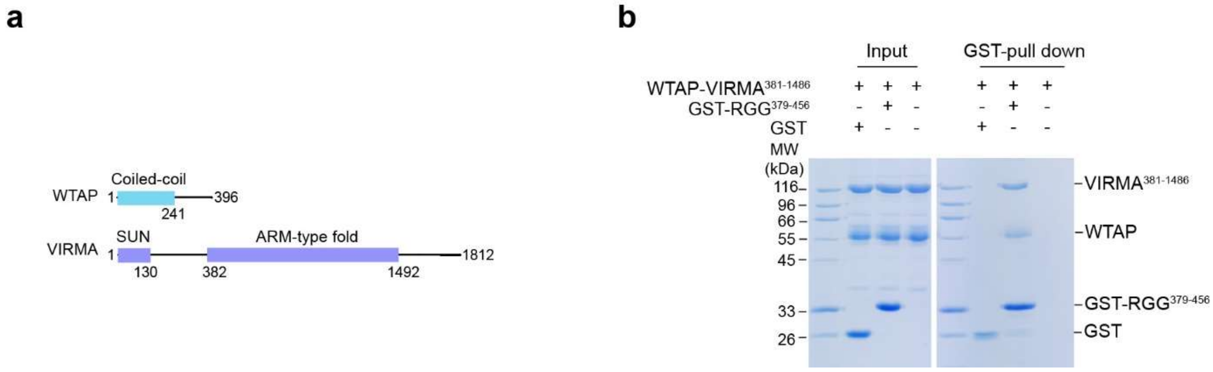

Fig. S7 GST pull-down assays to assess the interaction between WTAP-VIRMA$^{381\text{-}1486}$ and the RGG motif. a Schematic diagram for the domain information of WTAP and VIRMA. **b** The GST pull-down assay between GST-RGG$^{379\text{-}456}$ and WTAP-VIRMA$^{381\text{-}1486}$. WTAP-VIRMA$^{381\text{-}1486}$, a truncated form of WTAP-VIRMA with much higher yield and preserved ability to interact with the RGG motif.

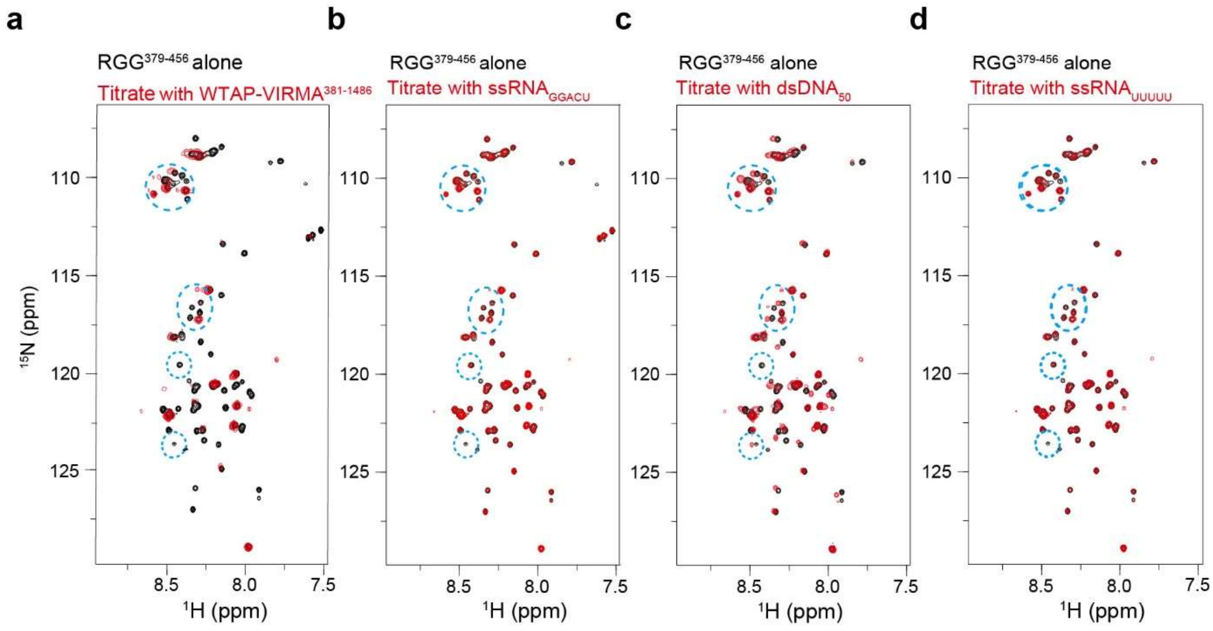

Fig. S8 NMR titrations of the RGG motif. a-d To conduct NMR titration experiments, WTAP-VIRMA$^{381\text{-}1486}$ (**a**), $ssRNA_{GGACU}$ (**b**), $dsDNA_{50}$ (**c**) or $ssRNA_{UUUUU}$ (**d**) were added into ^{15}N-labelled RGG (50 μM). The final concentrations of WTAP-VIRMA$^{381\text{-}1486}$, $ssRNA_{GGACU}$, $dsDNA_{50}$ and $ssRNA_{UUUUU}$ are 25 μM.
